# Comparative Impact of NSAIDs Versus Acetaminophen on Mortality in Stevens–Johnson Syndrome and Toxic Epidermal Necrolysis: A Retrospective Cohort Study of 2484 Patients From a Nationwide Inpatient Database

**DOI:** 10.1111/1346-8138.70082

**Published:** 2025-11-26

**Authors:** Rino Toyoshima, Shotaro Aso, Hiroki Matsui, Kiyohide Fushimi, Natsumi Hama, Riichiro Abe, Hideo Yasunaga, Shinichi Sato, Sayaka Shibata

**Affiliations:** ^1^ Department of Dermatology, Graduate School of Medicine The University of Tokyo Tokyo Japan; ^2^ Department of Health Services Research, Graduate School of Medicine The University of Tokyo Tokyo Japan; ^3^ Department of Clinical Epidemiology and Health Economics, School of Public Health The University of Tokyo Tokyo Japan; ^4^ Department of Health Policy and Informatics Institute of Science Tokyo Graduate School Tokyo Japan; ^5^ Division of Dermatology Graduate School of Medicine, Niigata University Graduate School of Medical and Dental Sciences Niigata Japan

**Keywords:** acetaminophen, chronic kidney disease (CKD), nonsteroidal anti‐inflammatory drugs (NSAIDs), Stevens–Johnson syndrome (SJS), toxic epidermal necrolysis (TEN)

## Abstract

Stevens–Johnson syndrome (SJS) and toxic epidermal necrolysis (TEN) are severe and life‐threatening mucocutaneous disorders, primarily triggered by medications. Despite the frequent need for antipyretic and analgesic therapy, the impact of nonsteroidal anti‐inflammatory drugs (NSAIDs) and acetaminophen on clinical outcomes in patients with SJS/TEN remains unclear. This study aimed to compare the effects of NSAIDs and acetaminophen use on in‐hospital mortality, infection‐related events, and renal outcomes, with particular attention to the presence or absence of chronic kidney disease (CKD). We conducted a retrospective analysis using a nationwide administrative database of over 1200 acute care hospitals in Japan between July 2010 and March 2022. Adult patients diagnosed with SJS or TEN who received either NSAIDs or acetaminophen within the first 5 days of hospitalization were included. Patients who received both drugs or neither were excluded. Among 8301 eligible patients, 2484 met inclusion criteria. Overall mortality did not differ significantly between groups (4.1% vs. 4.6%; risk difference [RD], −0.6%; 95% confidence interval [CI], −2.5% to 1.4%). In patients without CKD, NSAID use was associated with lower mortality (2.6% vs. 4.3%; RD, −1.7%; 95% CI, −3.4% to 0.0%). Conversely, in patients with CKD, acetaminophen use was associated with lower mortality (12.0% vs. 38.2%; RD, 26.2%; 95% CI, 5.0% to 47.4%). In conclusion, NSAID use may be associated with relatively improved survival compared with acetaminophen in patients without CKD, while acetaminophen appears safer in those with CKD, suggesting that renal function may inform the selection of antipyretic or analgesic therapy when such treatment is unavoidable. As both drugs are known causative agents of SJS/TEN, these results should be interpreted with caution. Further studies are warranted to validate these observational findings.

**Abbreviations**
ASD: absolute standardized difference
BMI: body mass index
CI: confidence intervals
CKD: chronic kidney disease
DPC: Diagnosis Procedure Combination
ICD‐10: *International Classification of Diseases, Tenth Revision*
ICU: Intensive Care Unit
JCS: Japan Come Scale
NSAIDs: nonsteroidal anti‐inflammatory drugs
RD: risk differences
SD: standard derivatives
SJS: Stevens–Johnson syndrome
TEN: Toxic epidermal necrolysis

## 1 Introduction

Stevens–Johnson syndrome (SJS) and toxic epidermal necrolysis (TEN) are severe mucocutaneous disorders mainly induced by medications, characterized by widespread blistering and epidermal detachment [1]. These conditions are classified based on the extent of epidermal detachment: SJS involves less than 10% of the body surface area, overlap SJS/TEN involves 10%–30%, and TEN involves more than 30% [1, 2, 3, 4, 5]. Widespread epidermal necrosis triggers dermal exposure [6], making patients highly susceptible to secondary infections, often leading to sepsis [7, 8]. Sepsis is the most common cause of death in SJS/TEN, with mortality rates of approximately 10% for SJS and up to 30% for TEN [2, 4, 5], emphasizing the clinical severity of these conditions [3, 5, 9, 10, 11, 12].

In addition to sepsis‐related risks, patients frequently experience severe pain and high‐grade fever [4, 7, 8, 13], which requires careful management to improve quality of life and outcomes [7, 8, 13]. Both the US and UK Guidelines recommend a stepwise approach for pain control, starting with acetaminophen and escalating to opioid‐based treatment as needed [8, 14]. Nonsteroidal anti‐inflammatory drugs (NSAIDs) are generally avoided to prevent potential risks to kidney function and gastric ulcers [8, 14]. Fever is another common and challenging symptom in SJS/TEN. Although fever may support immune responses, persistent high body temperature can worsen clinical outcomes in critically ill patients [15, 16, 17]. Studies in intensive care populations have shown that elevated temperature is associated with increased mortality risk, suggesting that proper fever control is essential [18, 19, 20, 21].

Despite these observations, no studies have directly compared the prognostic impact of NSAIDs and acetaminophen in SJS/TEN, and evidence‐based guidance on their use remains limited. Therefore, this study aimed to investigate the association between in‐hospital use of NSAIDs or acetaminophen and clinical outcomes in SJS/TEN. Since both NSAIDs and acetaminophen can act as culprit drugs for SJS/TEN and preadmission prescriptions are unavailable in the national database, we restricted our analysis to patients who received either NSAIDs only or acetaminophen only after admission. The findings should thus be interpreted as relative associations between the two options among treated patients rather than as evidence supporting the use of these drugs in all cases.

## 2 Method

### 2.1 Ethical Approval

The institutional review board of the University of Tokyo reviewed and approved the study protocol. Patient informed consent was waived because of the anonymous nature of the data. This study adhered to the tenets of the Declaration of Helsinki.

### 2.2 Data Source

This retrospective cohort study utilized routinely collected data from the Diagnosis Procedure Combination (DPC) inpatient database [22]. The database includes the following data: unique hospital identifiers, patient's sex, age, and body mass index (BMI) at admission; smoking history (including both current and former smoking), main diagnoses, comorbidities at admission, and complications after admission recorded with Japanese text data and using *International Classification of Diseases, Tenth Revision* (ICD‐10) codes; interventional/surgical procedures indexed by the original Japanese codes; length of stay; discharge status; and hospitalization costs. Hospitalization costs were based on reference prices in the Japanese national fee schedule for item‐by‐item prices for surgical, pharmaceutical, laboratory, and other inpatient services. All discharge abstract data for each patient were recorded at discharge by attending physicians. A previous validation study showed good sensitivity and specificity of the diagnoses and procedure records in the database [23]. Data were collected from more than 1200 acute care hospitals—approximately 90% of all tertiary care hospitals in Japan.

### 2.3 Patient Selection

The national database was searched for adult patients (≥ 15 years) hospitalized with a diagnosis of SJS (ICD‐10 code, L51.1) or TEN (ICD‐10 code, L51.2) from July 2010 through March 2022. Among these patients, those who were prescribed NSAIDs or acetaminophen within 5 days of hospitalization were included. This five‐day window was chosen to reflect symptomatic management during the early phase of hospitalization, rather than treatment for complications or prolonged fever. Because culprit drugs for SJS/TEN are typically discontinued upon hospitalization, prescriptions during this period were assumed to represent symptomatic treatment after disease onset rather than causative exposure. Therefore, cases in which NSAIDs or acetaminophen were suspected causative agents were considered effectively excluded from the analysis. Acetaminophen administration included oral, intravenous, and suppository forms, while NSAIDs included oral, intravenous, suppository, and intramuscular formulations. NSAIDs covered in this study included anthranilic acid derivatives, salicylic acid derivatives, pyrazole derivatives, phenylacetic acid derivatives, indole derivatives, propionic acid derivatives, and oxicams [24]. The initiation date of systemic NSAIDs or acetaminophen was defined as the index date.

### 2.4 Exposure

Patients who received NSAIDs were included in the NSAIDs group, and those who received acetaminophen were included in the acetaminophen group. Patients who received both acetaminophen and NSAIDs on the same day were excluded from the analysis. Drug exposure was defined based solely on in‐hospital prescriptions within the first 5 days after admission, as the national database does not contain information on prescriptions prior to hospitalization.

### 2.5 Outcomes

The primary outcome was in‐hospital mortality. Secondary outcomes included the development of infection defined as antibiotic use (penicillins, cephalosporins, fluoroquinolones, and carbapenems), the development of acute kidney diseases defined as receipt of hemodialysis (treatment after the fifth day of hospitalization), total cost, length of stay, and Intensive Care Unit (ICU) admission. Hemodialysis was evaluated considering the potential for renal impairment.

### 2.6 Statistical Analysis

Continuous variables are presented as mean and standard derivatives (SD), whereas categorical variables are presented as frequencies and percentages. We compared the crude outcomes between the NSAIDs and the acetaminophen groups using the *t*‐test for continuous outcomes and the *χ* ^2^ test for categorical outcomes.

To evaluate the outcomes, we used a propensity‐score overlap weighting method to estimate the adjusted outcomes. We calculated the propensity scores for receiving NSAIDs using a multivariable logistic regression analysis, which included the above covariates as the dependent variables [25, 26, 27, 28, 29, 30, 31, 32]. The patients receiving NSAIDs were weighted by 1‐ propensity score, while those receiving acetaminophen were weighted by propensity score. To assess the balance of patient characteristics between the groups, we calculated the absolute standardized differences for each covariate in the weighted and unweighted populations. A standardized difference of less than 10% was considered a negligible imbalance [33, 34]. After applying the overlap weights, we calculated the risk differences (RD) and their 95% confidence intervals (CI) in in‐hospital mortality, infection, and acute kidney diseases between the NSAIDs and acetaminophen groups. We used generalized linear models with identity link function to calculate the differences and their 95% CIs in total costs between the groups. We conducted subgroup analyses where the eligible patients were divided into (i) those with SJS and TEN, and (ii) those with and without chronic kidney disease (CKD). Chronic kidney disease (CKD) was defined based on ICD‐10 codes (N18) as comorbidity at admission. To test the robustness of the five‐day exposure definition, we conducted sensitivity analyses using alternative cutoff points at Day 2 and Day 3 of hospitalization. The two‐sided significance level for all tests was defined as *p* < 0.05. All analyses were performed from June 21, 2024 to September 11, 2024, using Stata/SE, version 18 (Stata Corp).

## 3 Results

### 3.1 Study Population

**FIGURE 1:**
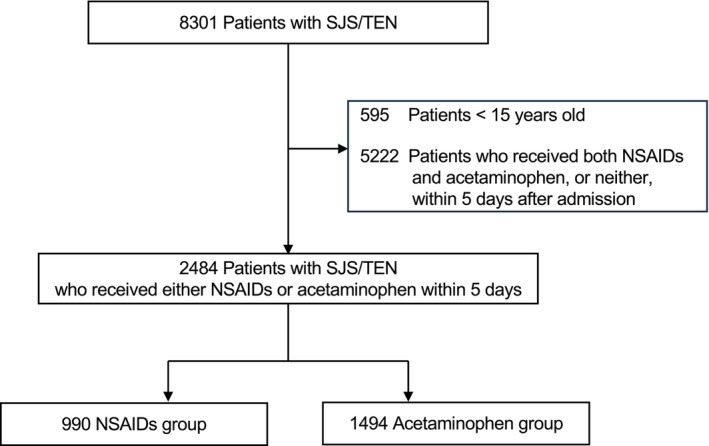
Flowchart of patient selection. Out of 8301 SJS/TEN patients, 595 under 15 years old and 5222 who received both NSAIDs and acetaminophen or neither within 5 days of admission were excluded. Of the remaining 2484 patients, 990 received NSAIDs first (NSAIDs group), and 1494 received acetaminophen first (Acetaminophen group) within five days of admission.

A total of 8301 patients with SJS/TEN were identified during the study period. We excluded 595 patients who were under 15 years old and 5222 patients who received both NSAIDs and acetaminophen or neither within 5 days after admission. Of the remaining 2484 patients, 990 received NSAIDs first (NSAIDs group), and 1494 received acetaminophen first (Acetaminophen group) within 5 days of the index date (Figure 1). Of note, 5222 patients (≈63%) who received both drugs or neither within 5 days were excluded by design to minimize exposure misclassification related to unobserved preadmission use.

### 3.2 Patient Sample and Baseline Characteristics

The characteristics of patients in the NSAIDs and acetaminophen groups are shown in Table 1. Among the NSAIDs group, 850 patients (85.9%) had SJS, while 140 patients (14.1%) had TEN. In the acetaminophen group, 1269 patients (84.9%) had SJS, and 225 patients (15.1%) had TEN. After overlap weighting, baseline characteristics were well balanced between the two groups.

**TABLE 1:** Patient characteristics before and after overlap weighting: NSAIDs versus Acetaminophen Group.

	Before overlap weighting			After overlap weighting		
	NSAIDs, *n* = 990	Acetaminophen, *n* = 1494	ASD^a^	NSAIDs	Acetaminophen	ASD^a^
**SJS/TEN, *n* (%)**			0.03			0.00
SJS	850 (85.9)	1269 (84.9)		(86.6)	(86.3)	
TEN	140 (14.1)	225 (15.1)		(13.4)	(13.7)	
**Age and sex**						
Age, years (SD)	56.7 (19.6)	59.0 (20.1)	0.12	57.6 (19.6)	57.7 (20.5)	0.01
Male, *n* (%)	462 (46.7)	726 (48.6)	0.04	(46.5)	(46.4)	0.00
**BMI, kg/m** ^**2**^, **n (%)**			0.10			0.00
< 18.5	120 (13.1)	227 (16.3)		(15.1)	(15.2)	
18.5–24.9	600 (65.6)	881 (63.2)		(64.1)	(63.9)	
25.0–29.9	159 (17.4)	224 (16.1)		(16.5)	(16.4)	
≥ 30.0	36 (3.9)	61 (4.4)		(4.4)	(4.4)	
**Smoking history, *n* (%)**			0.02			0.00
Current/past	251 (29.5)	365 (28.8)		(29.7)	(29.6)	
**JCS, *n* (%)**			0.12			0.02
0	940 (94.9)	1377 (92.2)		(94.4)	(94.4)	
1–3	45 (4.5)	99 (6.6)		(5.3)	(5.3)	
10–30	3 (0.3)	12 (0.8)		(0.3)	(0.3)	
100–300	2 (0.2)	6 (0.4)		0.0	0.0	
**ICU and emergency care, *n* (%)**	45 (4.5)	101 (6.8)	0.10	(5.3)	(5.4)	0.00
**Mechanical ventilation, *n* (%)**	9 (0.9)	24 (1.6)	0.06	(5.3)	(5.4)	0.00
**Charlson comorbidity index, *n* (%)**			0.07			0.01
0	702 (70.9)	1027 (68.7)		(69.0)	(68.8)	
1	95 (9.6)	135 (9.0)		(9.4)	(9.4)	
2	136 (13.7)	238 (15.9)		(15.9)	(15.9)	
≥ 3	57 (5.8)	94 (6.3)		(5.7)	(5.9)	
**Comorbidities, *n* (%)**						
Chronic kidney diseases	33 (3.3)	71 (4.8)	0.07	(4.2)	(4.1)	0.00
Cancer	98 (9.9)	169 (11.3)	0.05	(11.1)	(11.3)	0.01
Diabetes	103 (10.4)	209 (14.0)	0.11	(12.7)	(12.7)	0.00
Liver diseases	57 (5.8)	68 (4.6)	0.05	(5.3)	(5.4)	0.00
Autoimmune diseases	26 (2.6)	51 (3.4)	0.05	(3.3)	(3.3)	0.00
**Skin disease, *n* (%)**						
Atopic dermatitis	21 (2.1)	13 (0.9)	0.10	(1.4)	(1.3)	0.00
Psoriasis	5 (0.5)	5 (0.3)	0.03	(0.4)	(0.4)	0.01
Pemphigus	5 (0.5)	2 (0.1)	0.07	(0.3)	(0.2)	0.02
Bullous pemphigoid	5 (0.5)	5 (0.3)	0.03	(0.5)	(0.4)	0.01

*Note:* After overlap weighting, there was no difference in the characteristics of patients between the NSAIDs and acetaminophen groups.

^a^ ASD, absolute standardized difference (ASD of < 0.1 suggests adequate variable balance after propensity matching).

### 3.3 Overall Analysis

As shown in Table 2, the overall analysis revealed no significant difference between the NSAIDs and acetaminophen groups in terms of in‐hospital mortality (4.1% vs. 4.6%; RD, −0.6%; 95% CI, −2.5% to 1.4%; *p* = 0.58), the frequency of antibiotic use on or after the fifth day of hospitalization (7.5% vs. 7.4%; RD, 0.1%; 95% CI, −2.3% to 2.5%; *p* = 0.94), the rate of hemodialysis on or after the fifth day of hospitalization (0.8% vs. 1.0%; RD, −0.2%; 95% CI, −1.1% to 0.7%; *p* = 0.62), total costs (US$1 386 659 vs. $1 454 340; difference, −$63 628; 95% CI, −$193 167 to $65 911; *p* = 0.34), length of stay (18 days vs. 18 days; difference, −0.2 days; 95% CI, −2.2 days to 1.8 days; *p* = 0.85), and ICU admission (6.5% vs. 6.8%; RD, −0.4%; 95% CI, −2.7% to 1.9%; *p* = 0.75).

**TABLE 2:** Outcome analysis of entire SJS/TEN patients after propensity‐score overlap weighting: NSAIDs versus Acetaminophen Group.

Outcome	Treatment group		RD/difference	95% CI	*p*
	NSAIDs	Acetaminophen			
Mortality, %	4.1	4.6	−0.6	−2.5 to 1.4	0.58
Antibiotic use, %	7.5	7.4	0.1	−2.3 to 2.5	0.94
Hemodialysis use, %	0.8	1.0	−0.2	−1.1 to 0.7	0.62
Total cost, US$	1 386 659	1 454 340	−63 628	−193 167 to 65 911	0.34
Length of stay, days, median	18	18	−0.2	−2.2 to 1.8	0.85
ICU/emergency care admission, %	6.5	6.8	−0.4	−2.7 to 1.9	0.75

*Note:* The frequency of antibiotic use and hemodialysis use were based on data collected after the fifth day of hospitalization. There were no significant differences in mortality, antibiotic use, hemodialysis use, total cost, length of stay, and admission to ICU or emergency care between the NSAIDs and the acetaminophen group.

### 3.4 Subgroup Analyses by Disease Type

The subgroup analyses of SJS and TEN similarly showed no significant differences in outcomes between the NSAIDs and the acetaminophen group in in‐hospital mortality (SJS patients: 2.6% vs. 2.7%; RD, −0.1%; 95% CI, −1.8% to 1.5%; *p* = 0.86) (TEN patients: 13.8% versus 16.7%; RD −3.0%; 95% CI, −12.2% to 6.4%; *p* = 0.54) (Table 3). No significant differences were observed in secondary outcomes between these two groups.

**TABLE 3:** Subgroup analysis by SJS and TEN after propensity‐score overlap weighting: NSAIDs versus Acetaminophen Group.

Outcome	SJS					TEN				
	Treatment group		RD/difference	95% CI	*p*	Treatment group		RD/difference	95% CI	*p*
	NSAIDs	Acetaminophen				NSAIDs	Acetaminophen			
Mortality, %	2.6	2.7	−0.1	−1.8 to 1.5	0.86	13.8	16.7	−3.0	−12.2 to 6.4	0.54
Antibiotic use, %	6.2	6.0	0.2	−2.2 to 2.6	0.87	15.3	15.7	−0.4	−9.5 to 8.7	0.93
Hemodialysis use, %	0.5	0.4	0.2	−0.5 to 0.9	0.63	2.3	5.0	−2.7	−7.2 to 1.8	0.24
Total cost, US$	1 180 660	1 017 066	−1156	−114 895 to 112 582	0.98	2 030 983	2 581 005	−434 183	−978 695 to 110 329	0.12
Length of stay, days, median	16	17	0.4	−1.4 to 2.3	0.67	31	31	−3.7	−11.3 to 3.9	0.34
ICU/emergency care admission, %	4.8	4.5	0.4	−1.8 to 2.5	0.74	16.9	21.7	−4.8	−14,4 to 4.8	0.33

*Note:* The frequency of antibiotic use and hemodialysis use were based on data collected after the fifth day of hospitalization. Both in SJS and TEN patients, there were no significant differences in mortality, antibiotic use, hemodialysis use, total cost, length of stay, and admission to ICU or emergency care between the NSAIDs and the acetaminophen group.

### 3.5 Subgroup Analyses by Chronic Kidney Disease Status

A significant finding was observed in the analysis stratified by the status of CKD. As shown in Table 4, among patients without CKD, those in the NSAIDs group exhibited significantly lower in‐hospital mortality compared to the acetaminophen group (2.6% vs. 4.3%; RD, −1.7%; 95% CI, −3.4% to 0.0%; *p* = 0.045). In contrast, for patients with CKD, in‐hospital mortality was significantly higher in the NSAIDs group compared to the acetaminophen group (38.2% vs. 12.0%; RD, 26.2%; 95% CI, 5.0% to 47.4%; *p* = 0.015). There were no significant differences in secondary outcomes between the NSAIDs and acetaminophen groups, regardless of CKD status.

**TABLE 4:** Subgroup analysis by CKD status after propensity‐score overlap weighting: NSAIDs versus Acetaminophen Group.

Outcome	Without CKD					With CKD				
	Treatment group		RD/difference	95% CI	*p*	Treatment group		RD/difference	95% CI	*p*
	NSAIDs	Acetaminophen				NSAIDs	Acetaminophen			
Mortality, %	2.6	4.3	−1.7	−3.4 to −0.0	0.045	38.2	12.0	26.2	5.0 to 47.4	0.015
Antibiotic use, %	7.0	6.9	0.0	−2.3 to 2.4	0.98	19.0	17.6	1.4	−18.1 to 21.0	0.89
Hemodialysis use, %	0.6	0.8	−0.2	−1.0 to 0.6	0.58	5.0	5.5	−0.5	−11.8 to 10.9	0.94
Total cost, US$, SD	1 232 408	1 325 067	−95 064	−21 048 to 20 353	0.11	2 962 783	3 138 065	629 256	−840 066 to 2 098 579	0.40
Length of stay, days, median	17	17	−0.4	−2.3 to 1.6	0.72	26	21	3.2	−12.4 to 18.8	0.69
ICU/emergency care admission, %	5.8	6.4	−0.6	−2.9 to 1.6	0.59	21.2	16.4	4.7	−14.8 to 24.1	0.64

*Note:* Antibiotic and hemodialysis use frequency were based on data collected after the fifth day of hospitalization. In CKD patients, mortality was lower in the NSAIDs group, whereas in non‐CKD patients, it was lower in the acetaminophen group. There were no significant differences in antibiotic use, hemodialysis use, total cost, length of stay, and admission to ICU or emergency care between the NSAIDs and the acetaminophen group.

### 3.6 Sensitivity Analyses

When the exposure definition was restricted to drug administration at Day 2 or Day 3 of hospitalization, no significant differences in in‐hospital mortality or secondary outcomes were observed between the NSAIDs and acetaminophen groups (Tables S1 and S2).

## 4 Discussion

In this retrospective study, we investigated the impact of administering NSAIDs or acetaminophen in patients developing SJS/TEN, utilizing a nationwide inpatient database and overlap weighting method. Our analysis observed that approximately 30% of the registered SJS/TEN patients (2484 out of 8301) received either NSAIDs or acetaminophen within the 5 days of hospitalization. There were no significant differences in overall in‐hospital mortality, infection rate as indicated by the frequency of antibiotic use, renal impairment as evaluated by the rate of hemodialysis, hospitalization costs, length of stay, or ICU/emergency care admission between the NSAIDs and acetaminophen groups in the entire SJS/TEN population as well as the subgroups of SJS and TEN. Importantly, NSAIDs use was significantly associated with lower in‐hospital mortality in patients without CKD, whereas acetaminophen use was linked to lower mortality in those with CKD.

Although both US and UK guidelines for SJS/TEN discourage NSAID use in the management of pain due to the potential risk of renal dysfunction [8, 14], our retrospective study did not observe a significant difference in severe renal outcomes requiring dialysis between the NSAIDs and acetaminophen groups, suggesting that the avoidance of NSAIDs may not be universally necessary for all SJS/TEN patients, particularly those without existing renal impairments. Our analysis, however, was limited to severe renal outcomes and may not have captured milder dysfunction detectable only by laboratory testing [35].

The key finding of this study was the opposing mortality trends according to CKD status. In patients without CKD, NSAIDs may have improved survival through their anti‐inflammatory effects by inhibiting cyclooxygenase and reducing pro‐inflammatory prostaglandins [36, 37, 38, 39]. NSAIDs may thus reduce inflammation and tissue damage more effectively than acetaminophen, which lacks substantial anti‐inflammatory effects [40]. Whereas in patients with CKD, NSAIDs could have worsened systemic deterioration, resulting in higher mortality. Because infection and dialysis rates were similar between groups, mortality differences are more likely attributable to systemic rather than renal progression. This suggests that NSAID use should be considered with caution and tailored to renal function. Still, caution is warranted when using NSAIDs for fever management in SJS/TEN. Fever may play a protective role by promoting immune activation, and studies in patients with sepsis have reported that the use of antipyretics was associated with increased mortality [19]. Additionally, NSAIDs may promote bacterial adhesion at sites of epidermal detachment, increasing the risk of infection and delaying wound healing [41].

There are several limitations in our study. First, because preadmission prescription data were unavailable, comparison with patients who received neither NSAIDs nor acetaminophen was not feasible. The “nonuse” group may have included patients who recently took these drugs before admission, causing exposure misclassification. To minimize this risk, we analyzed patients who received either drug within 5 days of admission. Thus, our findings reflect relative in‐hospital associations rather than evidence supporting general use. Nonetheless, some misclassification bias may remain due to the database structure. Second, due to the lack of preadmission data, renal impairment was defined based on the need for dialysis, which may underestimate the incidence of mild to moderate renal dysfunction. Third, we cannot identify severe ocular complications in the database due to the reimbursement system. Nevertheless, the fact that only about 30% of patients received these drugs likely reflects clinicians' caution regarding potential causality and ocular complications. Fourth, the decision to administer NSAIDs or acetaminophen may have been influenced by the attending physician's subjective assessment of disease severity, introducing potential treatment‐selection bias. Finally, due to the nature of the study using a nationwide inpatient database in Japan, the generalizability to other countries or healthcare systems may be limited.

In conclusion, although no significant difference in overall in‐hospital mortality was observed between patients treated with NSAIDs and those treated with acetaminophen, subgroup analyses revealed that acetaminophen use was associated with lower mortality among patients with CKD, whereas NSAID use was associated with lower mortality among those without CKD. These findings suggest the importance of individualized treatment strategies in the acute management of SJS/TEN, taking into account renal function and overall patient condition.

## Funding

This work was supported by grants from the Ministry of Health, Labour and Welfare, Japan (23AA2003), the Ministry of Education, Culture, Sports, Science and Technology of Japan (24K11447), and the Japan Agency for Medical Research and Development (24gm6910016h0001).

## Conflicts of Interest

Riichiro Abe is an Editorial Board member of the *Journal of Dermatology* and a co‐author of this article. To minimize bias, Dr. Abe was excluded from all editorial decision‐making related to the acceptance of this article for publication. The other authors declare no conflicts of interest for this article.

## Supporting information


**Data S1:** jde70082‐sup‐0001‐supinfo.docx.

## Data Availability

The data that support the findings of this study are available on request from the corresponding author. The data are not publicly available due to privacy or ethical restrictions.
