# Supplementary material for: Comparative Impact of NSAIDs Versus Acetaminophen on Mortality in Stevens–Johnson Syndrome and Toxic Epidermal Necrolysis: A Retrospective Cohort Study of 2484 Patients From a Nationwide Inpatient Database
Source: J Dermatol. 2025 Nov 26;53(1):54–62. doi: 10.1111/1346-8138.70082 (PMC12784796; doi:10.1111/1346-8138.70082)
Supplement: Supplementary file 1 — Data S1: jde70082‐sup‐0001‐supinfo.docx. [file JDE-53-54-s001.docx]

**Supplementary Table 1. Sensitivity Analysis of Outcomes at Day 2 of Admission: NSAIDs vs. Acetaminophen After Propensity-Score Overlap Weighting**

|  | **Treatment Group** | |  |  |  |
| --- | --- | --- | --- | --- | --- |
| **Outcome** | **NSAIDs** | **Acetaminophen** | **RD/difference** | **95% CI** | **P value** |
| Mortality, % | 4.0 | 3.4 | 0.7 | -1.2 to 2.6 | 0.51 |
| Antibiotic use, % | 12.1 | 11.8 | 0.3 | -2.9 to 3.5 | 0.86 |
| Hemodialysis use, % | 2.2 | 1.8 | 0.4 | -1.1 to 1.8 | 0.62 |
| Total cost, US$ | 1,359,181 | 1,327,746 | -55690 | -189,002 to 77621 | 0.41 |
| Length of stay, days, median | 17 | 18 | -0.5 | -2.7 to 1.6 | 0.61 |
| ICU/emergency care admission, % | 5.5 | 6.0 | -0.5 | -2.7 to 1.8 | 0.68 |

The frequency of antibiotic use and hemodialysis use were based on data collected after the fifth day of hospitalization.

There were no significant differences in mortality, antibiotic use, hemodialysis use, total cost, length of stay, and admission to ICU or emergency care between the NSAIDs and the acetaminophen group.

**Supplementary Table 2. Sensitivity Analysis of Outcomes at Day 3 of Admission: NSAIDs vs. Acetaminophen After Propensity-Score Overlap Weighting**

|  | **Treatment Group** | |  |  |  |
| --- | --- | --- | --- | --- | --- |
| **Outcome** | **NSAIDs** | **Acetaminophen** | **RD/difference** | **95% CI** | **P value** |
| Mortality, % | 3.9 | 3.8 | 0.1 | -1.8 to 2.0 | 0.91 |
| Antibiotic use, % | 9.2 | 10.2 | -1.0 | -3.9 to 1.7 | 0.46 |
| Hemodialysis use, % | 1.6 | 1.6 | 0.0 | -1.2 to 1.2 | 0.96 |
| Total cost, US$ | 1,325,879 | 1,468,514 | -103,151 | -234297 to 27995 | 0.12 |
| Length of stay, days, median | 17 | 18 | -0.9 | -2.9 to 1.0 | 0.35 |
| ICU/emergency care admission, % | 6.0 | 6.8 | -0.8 | -3.1 to 1.4 | 0.47 |

The frequency of antibiotic use and hemodialysis use were based on data collected after the fifth day of hospitalization.

There were no significant differences in mortality, antibiotic use, hemodialysis use, total cost, length of stay, and admission to ICU or emergency care between the NSAIDs and the acetaminophen group.
